# Supplementary material for: HIVprotI: an integrated web based platform for prediction and design of HIV proteins inhibitors
Source: J Cheminform. 2018 Mar 9;10:12. doi: 10.1186/s13321-018-0266-y (PMC5845081; doi:10.1186/s13321-018-0266-y)
Supplement: Supplementary file 2 — Additional file 2. Source code of HIVProtI web server. [file 13321_2018_266_MOESM2_ESM.zip › HIVprotI_Source-code/hivprotI/weka-3-6-6/doc/weka/filters/unsupervised/attribute/Discretize.html]

Discretize


---


|  |  |  |
| --- | --- | --- |
| **Overview** \| **Package** \| **Class** \| **Tree** \| **Deprecated** \| **Index** \| **Help** \| **Weka's home** | |  |
| **PREV CLASS**   **NEXT CLASS** | **FRAMES**    **NO FRAMES**     **All Classes** |  |
| SUMMARY: NESTED \| FIELD \| CONSTR \| METHOD | DETAIL: FIELD \| CONSTR \| METHOD |  |


---


## weka.filters.unsupervised.attribute Class Discretize

```
java.lang.Object
  weka.filters.Filter
      weka.filters.unsupervised.attribute.PotentialClassIgnorer
          weka.filters.unsupervised.attribute.Discretize
```

**All Implemented Interfaces:**: java.io.Serializable, CapabilitiesHandler, OptionHandler, RevisionHandler, WeightedInstancesHandler, UnsupervisedFilter

**Direct Known Subclasses:**: PKIDiscretize

---

``` public class Discretize extends PotentialClassIgnorer implements UnsupervisedFilter, WeightedInstancesHandler ```

An instance filter that discretizes a range of numeric attributes in the dataset into nominal attributes. Discretization is by simple binning. Skips the class attribute if set.


Valid options are:

```
 -unset-class-temporarily
  Unsets the class index temporarily before the filter is
  applied to the data.
  (default: no)
```

```
 -B <num>
  Specifies the (maximum) number of bins to divide numeric attributes into.
  (default = 10)
```

```
 -M <num>
  Specifies the desired weight of instances per bin for
  equal-frequency binning. If this is set to a positive
  number then the -B option will be ignored.
  (default = -1)
```

```
 -F
  Use equal-frequency instead of equal-width discretization.
```

```
 -O
  Optimize number of bins using leave-one-out estimate
  of estimated entropy (for equal-width discretization).
  If this is set then the -B option will be ignored.
```

```
 -R <col1,col2-col4,...>
  Specifies list of columns to Discretize. First and last are valid indexes.
  (default: first-last)
```

```
 -V
  Invert matching sense of column indexes.
```

```
 -D
  Output binary attributes for discretized attributes.
```

**Version:**
:   $Revision: 6568 $

**Author:**
:   Len Trigg (trigg@cs.waikato.ac.nz), Eibe Frank (eibe@cs.waikato.ac.nz)

**See Also:**: Serialized Form

---

**Constructor Summary**

`Discretize()`
  
          Constructor - initialises the filter

`Discretize(java.lang.String cols)`
  
          Another constructor, sets the attribute indices immediately


| **Method Summary** | |
| --- | --- |
| `java.lang.String` | `attributeIndicesTipText()`             Returns the tip text for this property |
| `boolean` | `batchFinished()`             Signifies that this batch of input to the filter is finished. |
| `java.lang.String` | `binsTipText()`             Returns the tip text for this property |
| `java.lang.String` | `desiredWeightOfInstancesPerIntervalTipText()`             Returns the tip text for this property |
| `java.lang.String` | `findNumBinsTipText()`             Returns the tip text for this property |
| `java.lang.String` | `getAttributeIndices()`             Gets the current range selection |
| `int` | `getBins()`             Gets the number of bins numeric attributes will be divided into |
| `Capabilities` | `getCapabilities()`             Returns the Capabilities of this filter. |
| `double[]` | `getCutPoints(int attributeIndex)`             Gets the cut points for an attribute |
| `double` | `getDesiredWeightOfInstancesPerInterval()`             Get the DesiredWeightOfInstancesPerInterval value. |
| `boolean` | `getFindNumBins()`             Get the value of FindNumBins. |
| `boolean` | `getInvertSelection()`             Gets whether the supplied columns are to be removed or kept |
| `boolean` | `getMakeBinary()`             Gets whether binary attributes should be made for discretized ones. |
| `java.lang.String[]` | `getOptions()`             Gets the current settings of the filter. |
| `java.lang.String` | `getRevision()`             Returns the revision string. |
| `boolean` | `getUseEqualFrequency()`             Get the value of UseEqualFrequency. |
| `java.lang.String` | `globalInfo()`             Returns a string describing this filter |
| `boolean` | `input(Instance instance)`             Input an instance for filtering. |
| `java.lang.String` | `invertSelectionTipText()`             Returns the tip text for this property |
| `java.util.Enumeration` | `listOptions()`             Gets an enumeration describing the available options. |
| `static void` | `main(java.lang.String[] argv)`             Main method for testing this class. |
| `java.lang.String` | `makeBinaryTipText()`             Returns the tip text for this property |
| `void` | `setAttributeIndices(java.lang.String rangeList)`             Sets which attributes are to be Discretized (only numeric attributes among the selection will be Discretized). |
| `void` | `setAttributeIndicesArray(int[] attributes)`             Sets which attributes are to be Discretized (only numeric attributes among the selection will be Discretized). |
| `void` | `setBins(int numBins)`             Sets the number of bins to divide each selected numeric attribute into |
| `void` | `setDesiredWeightOfInstancesPerInterval(double newDesiredNumber)`             Set the DesiredWeightOfInstancesPerInterval value. |
| `void` | `setFindNumBins(boolean newFindNumBins)`             Set the value of FindNumBins. |
| `boolean` | `setInputFormat(Instances instanceInfo)`             Sets the format of the input instances. |
| `void` | `setInvertSelection(boolean invert)`             Sets whether selected columns should be removed or kept. |
| `void` | `setMakeBinary(boolean makeBinary)`             Sets whether binary attributes should be made for discretized ones. |
| `void` | `setOptions(java.lang.String[] options)`             Parses a given list of options. |
| `void` | `setUseEqualFrequency(boolean newUseEqualFrequency)`             Set the value of UseEqualFrequency. |
| `java.lang.String` | `useEqualFrequencyTipText()`             Returns the tip text for this property |

**Methods inherited from class weka.filters.unsupervised.attribute.PotentialClassIgnorer**

`getIgnoreClass, getOutputFormat, ignoreClassTipText, setIgnoreClass`

**Methods inherited from class weka.filters.Filter**

`batchFilterFile, filterFile, getCapabilities, isFirstBatchDone, isNewBatch, isOutputFormatDefined, makeCopies, makeCopy, numPendingOutput, output, outputPeek, toString, useFilter, wekaStaticWrapper`

**Methods inherited from class java.lang.Object**

`equals, getClass, hashCode, notify, notifyAll, wait, wait, wait`

**Constructor Detail**

### Discretize

```
public Discretize()
```

:   Constructor - initialises the filter

---


### Discretize

```
public Discretize(java.lang.String cols)
```

:   Another constructor, sets the attribute indices immediately

    **Parameters:**: `cols` - the attribute indices


**Method Detail**

### listOptions

```
public java.util.Enumeration listOptions()
```

:   Gets an enumeration describing the available options.

    :   **Specified by:**: `listOptions` in interface `OptionHandler` **Overrides:**: `listOptions` in class `PotentialClassIgnorer`
    :   **Returns:**: an enumeration of all the available options.

---


### setOptions

```
public void setOptions(java.lang.String[] options)
                throws java.lang.Exception
```

:   Parses a given list of options.


    Valid options are:

    ```
     -unset-class-temporarily
      Unsets the class index temporarily before the filter is
      applied to the data.
      (default: no)
    ```

    ```
     -B <num>
      Specifies the (maximum) number of bins to divide numeric attributes into.
      (default = 10)
    ```

    ```
     -M <num>
      Specifies the desired weight of instances per bin for
      equal-frequency binning. If this is set to a positive
      number then the -B option will be ignored.
      (default = -1)
    ```

    ```
     -F
      Use equal-frequency instead of equal-width discretization.
    ```

    ```
     -O
      Optimize number of bins using leave-one-out estimate
      of estimated entropy (for equal-width discretization).
      If this is set then the -B option will be ignored.
    ```

    ```
     -R <col1,col2-col4,...>
      Specifies list of columns to Discretize. First and last are valid indexes.
      (default: first-last)
    ```

    ```
     -V
      Invert matching sense of column indexes.
    ```

    ```
     -D
      Output binary attributes for discretized attributes.
    ```

    :   **Specified by:**: `setOptions` in interface `OptionHandler` **Overrides:**: `setOptions` in class `PotentialClassIgnorer`
    :   **Parameters:**: `options` - the list of options as an array of strings **Throws:**: `java.lang.Exception` - if an option is not supported

---


### getOptions

```
public java.lang.String[] getOptions()
```

:   Gets the current settings of the filter.

    :   **Specified by:**: `getOptions` in interface `OptionHandler` **Overrides:**: `getOptions` in class `PotentialClassIgnorer`
    :   **Returns:**: an array of strings suitable for passing to setOptions

---


### getCapabilities

```
public Capabilities getCapabilities()
```

:   Returns the Capabilities of this filter.

    :   **Specified by:**: `getCapabilities` in interface `CapabilitiesHandler` **Overrides:**: `getCapabilities` in class `Filter`
    :   **Returns:**: the capabilities of this object **See Also:**: `Capabilities`

---


### setInputFormat

```
public boolean setInputFormat(Instances instanceInfo)
                       throws java.lang.Exception
```

:   Sets the format of the input instances.

    :   **Overrides:**: `setInputFormat` in class `PotentialClassIgnorer`
    :   **Parameters:**: `instanceInfo` - an Instances object containing the input instance structure (any instances contained in the object are ignored - only the structure is required). **Returns:**: true if the outputFormat may be collected immediately **Throws:**: `java.lang.Exception` - if the input format can't be set successfully

---


### input

```
public boolean input(Instance instance)
```

:   Input an instance for filtering. Ordinarily the instance is processed
    and made available for output immediately. Some filters require all
    instances be read before producing output.

    :   **Overrides:**: `input` in class `Filter`
    :   **Parameters:**: `instance` - the input instance **Returns:**: true if the filtered instance may now be collected with output(). **Throws:**: `java.lang.IllegalStateException` - if no input format has been defined.

---


### batchFinished

```
public boolean batchFinished()
```

:   Signifies that this batch of input to the filter is finished. If the
    filter requires all instances prior to filtering, output() may now
    be called to retrieve the filtered instances.

    :   **Overrides:**: `batchFinished` in class `Filter`
    :   **Returns:**: true if there are instances pending output **Throws:**: `java.lang.IllegalStateException` - if no input structure has been defined

---


### globalInfo

```
public java.lang.String globalInfo()
```

:   Returns a string describing this filter

    :   **Returns:**: a description of the filter suitable for displaying in the explorer/experimenter gui

---


### findNumBinsTipText

```
public java.lang.String findNumBinsTipText()
```

:   Returns the tip text for this property

    :   **Returns:**: tip text for this property suitable for displaying in the explorer/experimenter gui

---


### getFindNumBins

```
public boolean getFindNumBins()
```

:   Get the value of FindNumBins.

    :   **Returns:**: Value of FindNumBins.

---


### setFindNumBins

```
public void setFindNumBins(boolean newFindNumBins)
```

:   Set the value of FindNumBins.

    :   **Parameters:**: `newFindNumBins` - Value to assign to FindNumBins.

---


### makeBinaryTipText

```
public java.lang.String makeBinaryTipText()
```

:   Returns the tip text for this property

    :   **Returns:**: tip text for this property suitable for displaying in the explorer/experimenter gui

---


### getMakeBinary

```
public boolean getMakeBinary()
```

:   Gets whether binary attributes should be made for discretized ones.

    :   **Returns:**: true if attributes will be binarized

---


### setMakeBinary

```
public void setMakeBinary(boolean makeBinary)
```

:   Sets whether binary attributes should be made for discretized ones.

    :   **Parameters:**: `makeBinary` - if binary attributes are to be made

---


### desiredWeightOfInstancesPerIntervalTipText

```
public java.lang.String desiredWeightOfInstancesPerIntervalTipText()
```

:   Returns the tip text for this property

    :   **Returns:**: tip text for this property suitable for displaying in the explorer/experimenter gui

---


### getDesiredWeightOfInstancesPerInterval

```
public double getDesiredWeightOfInstancesPerInterval()
```

:   Get the DesiredWeightOfInstancesPerInterval value.

    :   **Returns:**: the DesiredWeightOfInstancesPerInterval value.

---


### setDesiredWeightOfInstancesPerInterval

```
public void setDesiredWeightOfInstancesPerInterval(double newDesiredNumber)
```

:   Set the DesiredWeightOfInstancesPerInterval value.

    :   **Parameters:**: `newDesiredNumber` - The new DesiredNumber value.

---


### useEqualFrequencyTipText

```
public java.lang.String useEqualFrequencyTipText()
```

:   Returns the tip text for this property

    :   **Returns:**: tip text for this property suitable for displaying in the explorer/experimenter gui

---


### getUseEqualFrequency

```
public boolean getUseEqualFrequency()
```

:   Get the value of UseEqualFrequency.

    :   **Returns:**: Value of UseEqualFrequency.

---


### setUseEqualFrequency

```
public void setUseEqualFrequency(boolean newUseEqualFrequency)
```

:   Set the value of UseEqualFrequency.

    :   **Parameters:**: `newUseEqualFrequency` - Value to assign to UseEqualFrequency.

---


### binsTipText

```
public java.lang.String binsTipText()
```

:   Returns the tip text for this property

    :   **Returns:**: tip text for this property suitable for displaying in the explorer/experimenter gui

---


### getBins

```
public int getBins()
```

:   Gets the number of bins numeric attributes will be divided into

    :   **Returns:**: the number of bins.

---


### setBins

```
public void setBins(int numBins)
```

:   Sets the number of bins to divide each selected numeric attribute into

    :   **Parameters:**: `numBins` - the number of bins

---


### invertSelectionTipText

```
public java.lang.String invertSelectionTipText()
```

:   Returns the tip text for this property

    :   **Returns:**: tip text for this property suitable for displaying in the explorer/experimenter gui

---


### getInvertSelection

```
public boolean getInvertSelection()
```

:   Gets whether the supplied columns are to be removed or kept

    :   **Returns:**: true if the supplied columns will be kept

---


### setInvertSelection

```
public void setInvertSelection(boolean invert)
```

:   Sets whether selected columns should be removed or kept. If true the
    selected columns are kept and unselected columns are deleted. If false
    selected columns are deleted and unselected columns are kept.

    :   **Parameters:**: `invert` - the new invert setting

---


### attributeIndicesTipText

```
public java.lang.String attributeIndicesTipText()
```

:   Returns the tip text for this property

    :   **Returns:**: tip text for this property suitable for displaying in the explorer/experimenter gui

---


### getAttributeIndices

```
public java.lang.String getAttributeIndices()
```

:   Gets the current range selection

    :   **Returns:**: a string containing a comma separated list of ranges

---


### setAttributeIndices

```
public void setAttributeIndices(java.lang.String rangeList)
```

:   Sets which attributes are to be Discretized (only numeric
    attributes among the selection will be Discretized).

    :   **Parameters:**: `rangeList` - a string representing the list of attributes. Since the string will typically come from a user, attributes are indexed from 1. eg: first-3,5,6-last **Throws:**: `java.lang.IllegalArgumentException` - if an invalid range list is supplied

---


### setAttributeIndicesArray

```
public void setAttributeIndicesArray(int[] attributes)
```

:   Sets which attributes are to be Discretized (only numeric
    attributes among the selection will be Discretized).

    :   **Parameters:**: `attributes` - an array containing indexes of attributes to Discretize. Since the array will typically come from a program, attributes are indexed from 0. **Throws:**: `java.lang.IllegalArgumentException` - if an invalid set of ranges is supplied

---


### getCutPoints

```
public double[] getCutPoints(int attributeIndex)
```

:   Gets the cut points for an attribute

    :   **Parameters:**: `attributeIndex` - the index (from 0) of the attribute to get the cut points of **Returns:**: an array containing the cutpoints (or null if the attribute requested has been discretized into only one interval.)

---


### getRevision

```
public java.lang.String getRevision()
```

:   Returns the revision string.

    :   **Specified by:**: `getRevision` in interface `RevisionHandler` **Overrides:**: `getRevision` in class `Filter`
    :   **Returns:**: the revision

---


### main

```
public static void main(java.lang.String[] argv)
```

:   Main method for testing this class.

    :   **Parameters:**: `argv` - should contain arguments to the filter: use -h for help


---


|  |  |  |
| --- | --- | --- |
| **Overview** \| **Package** \| **Class** \| **Tree** \| **Deprecated** \| **Index** \| **Help** \| **Weka's home** | |  |
| **PREV CLASS**   **NEXT CLASS** | **FRAMES**    **NO FRAMES**     **All Classes** |  |
| SUMMARY: NESTED \| FIELD \| CONSTR \| METHOD | DETAIL: FIELD \| CONSTR \| METHOD |  |


---
